# Structural Modification of Polymers Functionalized with Mango Leaf Extract by Supercritical Impregnation: Approaching of Further Food and Biomedical Applications

**DOI:** 10.3390/polym14122413

**Published:** 2022-06-14

**Authors:** Cristina Cejudo-Bastante, Lidia Verano-Naranjo, Noemí Toro-Barrios, Clara Pereyra, Casimiro Mantell, Lourdes Casas

**Affiliations:** Chemical Engineering and Food Technology Department, Wine and Agrifood Research Institute (IVAGRO), University of Cadiz, Avda. República Saharaui, s/n, 11510 Puerto Real, Spain; noemi.torobarrios@alum.uca.es (N.T.-B.); clara.pereyra@uca.es (C.P.); casimiro.mantell@uca.es (C.M.); lourdes.casas@uca.es (L.C.)

**Keywords:** supercritical impregnation, functionalized polymers, TPU, PETG, mango leaf extract

## Abstract

Identifying new polymers from natural resources that can be effectively functionalized can have a substantial impact on biomedical devices and food preservation fields. Some of these polymers would be made of biodegradable, renewable and compostable materials, and present the kind of porosity required to effectively carry active compounds that confer on them the desired properties for their intended applications. Some natural extracts, such as mango leaf extract, have been proven to have high levels of antioxidant, antimicrobial or anti-inflammatory properties, making them good candidates for controlled-release applications. This work intends to investigate the supercritical impregnation of different types of polymers (ABS, PETG, TPU, PC and PCL) with mango leaf extract. The influence of temperature and pressure on the polymers’ structure (swelling and foaming processes) and their different behaviors have been analyzed. Thus, TPU and PC experience minimal structural modifications, while PETG, PCL and ABS, on the other hand, suffer quite significant structural changes. TPU and PETG were selected as the representative polymers for each one of these behaviors to delve into mango leaf extract impregnation processes. The bioactive capacity of the extract is present in either impregnated polymer, with 25.7% antioxidant activity by TPU processed at 35 °C and 100 bar and 32.9% antioxidant activity by PETG impregnated at 75 °C and 400 bar.

## 1. Introduction

Most conventional methods to incorporate active compounds into polymeric matrices are mainly based on the addition of these substances during the reactions that give rise to the polymer formation, during shaping or machining operations, or by the subsequent soaking of the polymeric element into an organic solvent that contains the substances that are to be impregnated into the polymer. These traditional methods present a number of drawbacks, some of them quite relevant, such as the thermal degradation of the active substances because of the high temperatures or the use of organic solvents that are not easy to remove from the final product [1]. Supercritical Solvent Impregnation (SSI) allows one to overcome the aforementioned drawbacks. This is one of the most groundbreaking technologies used to bind molecules into polymers. Supercritical carbon dioxide (scCO_2_) is used as the mobile phase to dissolve, diffuse and integrate the active substances into the polymer. The impregnation takes place in three stages [2]: (i) dissolution of the active substance in the supercritical fluid, (ii) swelling of the polymer matrix and diffusion of the active compound and (iii) depressurization and release of the CO_2_ in its gaseous form, while the active molecules get trapped inside the polymer. Operating temperatures are moderate (as carbon dioxide reaches supercritical conditions at just above 31 °C) and organic solvent are not needed. Sometimes small amounts of organic solvents are used to improve the process, but they do not come into direct contact with the polymer, which results in a final device that is free from any residual solvent.

Depending on the operating conditions and the polymer’s nature, direct contact with scCO_2_ may alter the internal structure or result in a permanent swelling of the polymer. For this reason, the depressurizing phase is crucial with regards to the final porosity of the polymer after it has been impregnated. Thus, an abrupt depressurization of the chamber when the polymer is saturated with CO_2_ may lead to an expansion of the polymer that would increase the porosity of the polymer [3]. Sometimes experiments that have been carried out by supercritical impregnation have focused on minimizing the modifications of polymer properties, and those that alter their structure more severely have been discarded. Nevertheless, polymers are very versatile matrices that can be employed in numerous applications depending on their structure. Certain significant changes resulting from supercritical methodologies do not imply that they become inevitably unsuitable for specific applications. For instance, when impregnated polymers were intended to be used as food preserving packaging, they should remain mostly unaltered and, therefore, any microbubbles or breakages might compromise their functionality over the storage time. The same polymer in the form of aerogels [4] or foams [5,6,7] could be employed as an effective release device rather than a packaging film. When such polymers are used in the biomedical field, they can perfectly operate as bioactive catheters or stents [8] as long as they maintain their structure, or even as functionalized scaffolds [9,10] when they present a high porosity ratio. In this sense, porosity is a crucial parameter that may drastically condition the final effective usage of a particular polymer.

With regard to the extracts obtained from *Mangifera indica* leaves, they have extensively demonstrated their nutraceutical, antioxidant and antimicrobial properties and, therefore, there suitability for different applications [11]. Thanks to their good affinity with CO_2_ and with certain polymeric matrices, they have been successfully used to impregnate active food packaging films [12,13], wound dressings or biomedical implants [8,14] with successful in vitro and in vivo tests. In a recently published previous work [15], the pro-angiogenic, anti-proliferative and antiapoptotic effect of mango leaves extract on endothelial colony-forming cells was demonstrated, making it a good candidate for further study as an additive for biomedical implants. However, one of the most important issues when determining the suitability of a functionalized polymer for a particular application is its migration kinetics, and this aspect has not yet been determined. The release kinetics of a particular active compound from a polymeric device into a fluid medium depends on multiple parameters, among which the diffusion of the solute, the swelling of the polymeric matrix in contact with the fluid and the degradability of the material stand out as the main mechanisms that control the process [16]. These three parameters are influenced by the porous structure of the polymer resulting from the contact with scCO_2_. This means that the operating conditions of the supercritical impregnation process have a direct influence on the way the active substance is released [17].

The present work focuses on a comparative assessment of the application of supercritical impregnation on different polymers of interest for biomedicine and food preservation purposes. The effect of supercritical carbon dioxide has been determined on acrylonitrile butadiene styrene (ABS), polyethylene terephthalate glycol (PETG), thermoplastic polyurethane (TPU), polycarbonate (PC) and polycaprolactone (PCL). According to the results obtained, two polymers were selected to study supercritical impregnation with MLE: PETG as a polymer that presents a high degree of swelling and TPU as an example of a polymer that does not present considerable swelling. Thus, the effect of pressure and temperature on the amount of impregnated extract and the antioxidant capacity of the generated material have been determined. In addition, the release kinetics of the impregnated active substance into a saline medium and its fitting to certain traditional drug delivery mathematical models (zero-order, first-order, Korsmeyer-Peppas and Higuchi) have been investigated.

## 2. Materials and Methods

### 2.1. Raw Material and Polymers

*Mangifera indica* L. leaves (Ken variety) were furnished by the Institute for subtropical and Mediterranean horticulture “La Mayora” (CSIC-UMA, Málaga, Spain).

The polymers used in the experiments were Acrylonitrile Butadiene Styrene (ABS) (Zhuhai Sunlu Industrial Co., Ltd. (Guangdong, China); Tg: 110 °C); Polyethylene Terephthalate-Glicol (PETG) (Amazon (Seattle, WA, USA), Tg: 85 °C); Thermoplastic Polyurethane (TPU) (Geeetech (Shenzhen, China), Tg: 80 °C); Polycarbonate (PC) (Prima (Malcöm, Switzerland), Tg: 150 °C); Polycaprolactone (PCL) (Daraz (Watertown, Pakistan), Tg: −60 °C).

Carbon dioxide from Abello Linde S.A. (Barcelona, Spain) (99.99% purity) was used for the impregnation experiments. The phosphate-buffered saline (PBS) reagents, dimethyl sulfoxide (DMSO) and dichloromethane (CH_2_Cl_2_) were supplied by Panreac AppliChem (Darmstadt, Germany). The partially denatured ethanol (96%) used to obtain M. indica L. extract were provided by Alcoholes del Sur (Córdoba, Spain). The 2,2-diphenyl-1-picrylhydrazyl reagent (DPPH) was purchased from Sigma-Aldrich (Steinheim, Germany).

### 2.2. Mango Leaf Extract Production

The mango leaves extract (MLE) used for the experiments was produced by Pressurized Liquid Extraction method (PLE) following the procedure published in a previous work by Rosales et al., 2021 [8].

The extraction was carried out by means of a supercritical extraction equipment provided by Thar Technologies (model SF1000, Pittsburgh, PA, USA) equipped with a one-liter capacity extractor. A filter paper cartridge containing approximately 500 g of previously crushed mango leaves and approximately 500 mL of ethanol was placed inside the extractor’s chamber. Then, the system temperature was set up at constant 80 °C and CO_2_ was injected until 200 bar was reached. After 12 h of operation, an extract with a dry weight concentration of 90 g/L was obtained. It was stored at 4 °C until further use.

### 2.3. Supercritical Impregnation Procedure

The experiments were carried out in a lab-scale high-pressure equipment provided by Thar Technologies (Pittsburgh, PA, USA). This was comprised of a condenser, a P50 high-pressure pump, a pre-heater, a 100 mL vessel with a thermostatic jacket and a back-pressure regulator (BPR). All the units were monitored and controlled. The impregnation experiments were conducted in batch mode following the procedure described in a previous work by Verano Naranjo et al., 2021 [17]. Two runs of experiments were completed. The first one involved the evaluation of the effect from the carbon dioxide at each specific supercritical conditions on the different polymer structures. Based on the results from this first run of experiments, two of the polymers were chosen as the most representative. The second run of experiments consisted of the supercritical impregnation of these two polymers with MLE under different processing conditions, as explained below.

#### 2.3.1. Evaluation of the Swelling Effect

The experiment consisted in introducing two pieces of 30 mm of a polymer filament into the impregnation vessel, heat and pump CO_2_ until the setpoint conditions are reached and, after the considered impregnation time has passed, depressurize the system. Experimental design 3^2^ has been carried out by varying pressure (100, 250 and 400 bar) and temperature (35, 55 and 75 °C) conditions in a wide range. The impregnation time was setup at 30 min and 100 bar/min was the depressurization rate applied. These conditions were selected considering a previous work [17]. The volumetric expansion or the swelling effect (S (%)) experienced by each sample was evaluated by measuring the difference of the filament diameter before ($d_{i}$) and after ($d_{f}$) the experiment (Equation (1)). All the experiments were completed four times.

(1) $$S\;\left(\%\right)=\frac{d_{f}-d_{i}}{d_{i}}\times 100=\left(\;\frac{d}{d_{i}}-1\right)\times 100$$

A cluster analysis was applied to the data on the swelling of the five polymers analyzed in order to determine if any subdivisions or groups (clusters) containing similar elements could be detected.

The hierarchical method was used, which initially considers as many groups as there are cases. In the following phase, the two closest groups are joined together. The process is repeated until a single group is formed. The square of the Euclidean distance was used as the measure of the proximity between cases or groups of cases. The statistical data were processed by means of the application Statgraphics centurion XIX.

#### 2.3.2. Impregnation with MLE

Based on the swelling effect obtained, two of the polymers were selected to be impregnated with MLE by supercritical solvent impregnation. One of them (TPU) had barely swollen under the supercritical conditions and the other one (PETG) had exhibited a more pronounced swelling.

This time, 3 mL of the extract with a concentration of 90 g/L was introduced in the impregnation vessel, together with the filaments, while avoiding any direct physical contact with the extract. For this purpose, a metal basket was employed. The experiment was realized in batch mode to guarantee a constant MLE concentration in the scCO_2_ phase at the impregnation conditions. The impregnation time was fixed at 2 h, according to previous works [8]. Pressure and temperature influences were studied, following a 2^2^ experimental design with two replicates, for the extreme values of pressure (100 and 400 bar) and temperature (35 and 75 °C) taken above.

The impregnation loadings were calculated spectrophotometrically by dissolving 50 mg of the impregnated polymer into 5 mL of an organic solvent (DMSO in the case of TPU and CH_2_Cl_2_ in the case of PETG). The absorbance of the solution was measured at 360 nm for TPU-DMSO and at 400 nm for PETG-CH_2_Cl_2_, and the loading was quantified by means of two calibration curves (Equations (2) and (3)) calculated for different MLE concentrations (between 10 and 300 mg/L).

(2) $$\mathrm{MLE}\;\mathrm{in}\;\mathrm{DMSO}:\;\mathrm{Abs}\left(360\;\mathrm{nm}\right)=0.0036\times \left[\mathrm{MLE}\right]\left(\mathrm{mg}/L\right)-0.0063;\;\;R^{2}=0.9997$$

(3) $${\mathrm{MLE}\;\mathrm{in}\;\mathrm{CH}}_{2}{\mathrm{Cl}}_{2}:\;\mathrm{Abs}\left(400\;\mathrm{nm}\right)=0.0080\times \left[\mathrm{MLE}\right]\left(\mathrm{mg}/L\right)-0.0583;R^{2}=0.9989$$

### 2.4. Scanning Electron Microscopy

The selected polymers (TPU and PETG) in the conditions that exhibited the largest loadings were visualized by Scanning Electron Microscopy (SEM) using a Nova NanoSEM 450 (FEI Company, Hillsboro, OR, USA) after covering them with a 10 nm gold coating under a 5 KV voltage. The changes in the polymers were observed both on the outer surface and on the surface of their cross-sections.

### 2.5. Determination of Antioxidant Capacity Using the DPPH Method

The antioxidant activity of the MLE was evaluated by means of a 2,2-diphenyl-1-picrylhydrazyl radical (DPPH*) assay following the method described by Brand-Williams and coworkers [18] For such an analysis, aliquots (0.1 mL) of MLE at different concentrations were added to 3.9 mL of a 6 × 10^−5^ mol/L DPPH ethanolic solution. After two hours, when the oxidative reaction had reached a steady state, the absorbance of the DPPH was measured.

The percentage of oxidation inhibition (OI) of each MLE sample was calculated according to Equation (4), where $Abs_{o}$ is the initial absorbance of the DPPH reagent and $Abs_{f}$ is the absorbance of the sample after the reaction.

(4) $$\mathrm{OI}\;\left(\%\right)=\frac{Abs_{i}-Abs_{f}}{Abs_{i}}\times 100$$

The correlation curve between the OI of the extract at different final concentration (between 2.5 and 37.5 mg/L) is calculated by means of Equation (5):

(5) $$\mathrm{OI}\;\left(\%\right)=-0.1062\;{\left[\mathrm{MLE}\right]}^{2}+6.4235\;\left[\mathrm{MLE}\right]\;;\;\;\;\;R^{2}=0.9992$$

The antioxidant capability of the filaments impregnated with the active substance was then determined. For this purpose, 50 mg of impregnated polymer was submerged in 10 mL of phosphate buffer saline (PBS) pH 7.4 and maintained at 37 °C to favor the diffusion of the extract for 7 days. Then, the antioxidant capacity of the polymer was determined by mixing 0.1 mL of that solution with 3.9 mL of a 6 × 10^−5^ mol/L DPPH ethanolic solution. It was then allowed to react for 2 h, and the oxidation inhibition was measured in a similar way as that of the extract. These measurements were carried out in quadruplicate.

### 2.6. In-Vitro MLE Release Analysis

The release kinetics of the MLE-impregnated polymers into a saline medium was determined by UV-VIS spectrophotometry. For this measurement, 50 mg of impregnated TPU or PETG were submerged into 5 mL of PBS pH 7.4 and maintained at 37 °C. An aliquot of the solution was regularly taken for spectrophotometric analysis and then put back into the rest of the solution. Its absorbance was measured at 275 nm, where the extract presents a peak due to the presence of polyphenols. The released MLE was quantified by means of a calibration curve (Equation (6)) generated at different extract concentrations in a PBS medium at between 1 and 70 mg/L.

(6) $$\mathrm{Abs}\left(275\;\mathrm{nm}\right)=0.0102\times \left[\mathrm{MLE}\right]\left(\mathrm{mg}/L\right)+0.032\;;{\;R}^{2}=0.9988$$

The extract released ratio at time t ($Q_{t}$) was calculated as the division of the accumulative mass of extract released at a certain time t ($m_{t}$) by the total mass of extract loaded or released at an infinite time ($m_{\infty }$):

(7) $$Q_{t}=\frac{m_{t}}{m_{\infty }}\;$$

## 3. Results

### 3.1. Swelling of Polymers under Supercritical CO_2_

**Figure 1:**
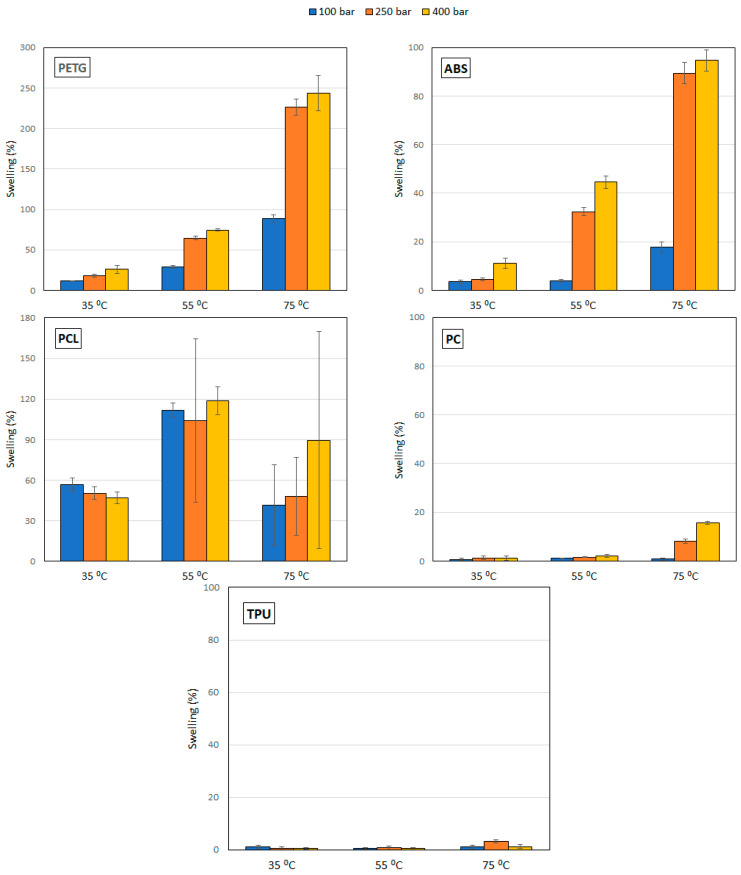
Swelling of the polymers under supercritical conditions of pressure and temperature.

The results of the permanent swelling of the polymers when in contact with CO_2_ under supercritical conditions are shown in Figure 1. In general, two different behaviors can be observed. On one hand, TPU and PC barely modified their volume. On the other hand, some polymers exhibited a high swelling percentage, like in the case of PETG, followed by ABS and PCL. Regarding the effect of pressure, in practically all the cases an increment of the pressure results in an enhancement of the swelling effect. Such enhancement is much more pronounced when the pressure was increased from 100 bar to 250 bar than when it was increased from 250 bar to 400 bar. In the PCL and TPU samples this behavior was not clearly observed. Regarding the effect of temperature, an enhancement of the swelling was observed with increasing temperature. In the case of PETG and PC, such greater swelling was much more pronounced when the temperature was increased from 55 to 75 °C than when it was increased from 35 to 55 °C.

**Figure 2:**
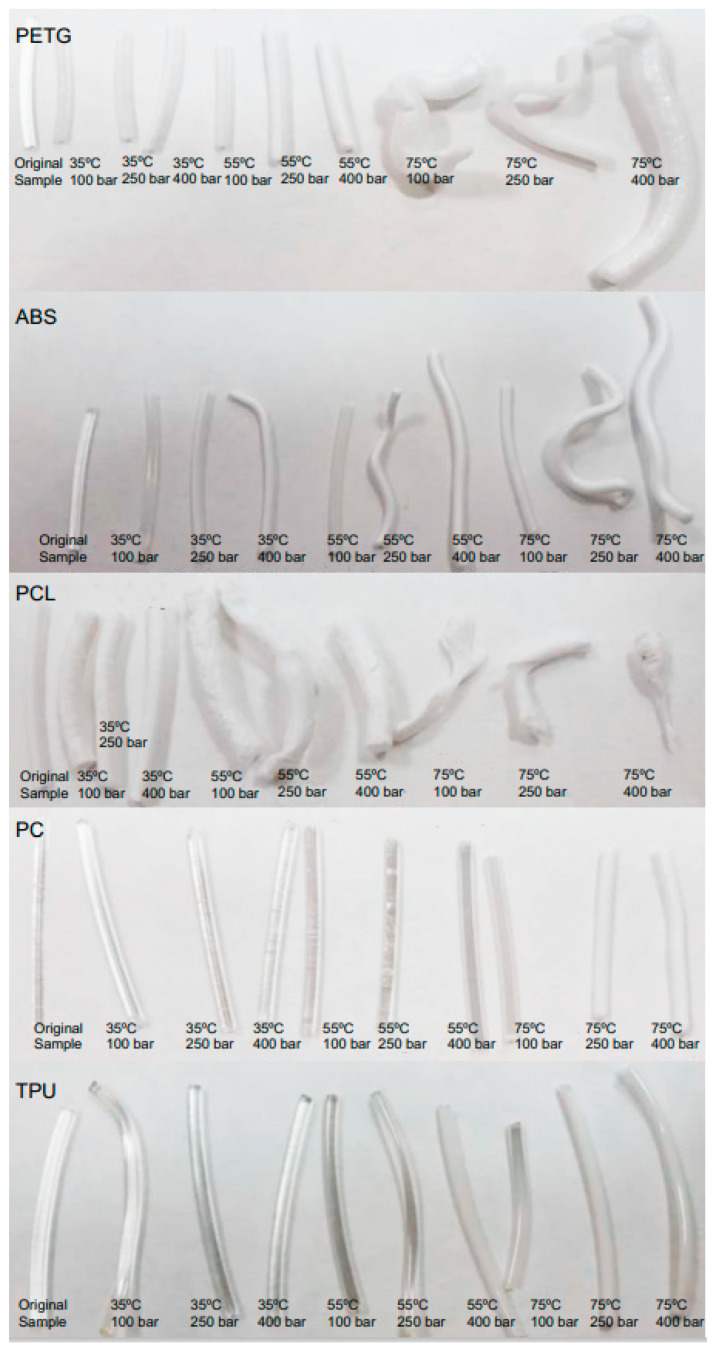
Picture of the polymers under different supercritical conditions.

Figure 2 includes some photographs of the polymers after being in contact with carbon dioxide under supercritical conditions. Photographs of the initial conformation of the polymers have also been included. The analysis of these images confirms the results presented in Figure 1, where some of the polymers, such as TPU and PC, do not practically change their structure with pressure and temperature variations, while others, on the other hand, deform significantly, especially with an increment of the operating temperature (PETG, ABS and PCL). PCL is, in particular, so drastically deformed at high temperature that the measuring of the polymer filament diameter becomes impossible. In this sense, and given its low melting point, it could only be accurately tested at 35 °C, while large deviations of the measurement could be observed in Figure 1 at 55 and 75 °C as the sample melted or irregularly swelled at certain points.

**Figure 3:**
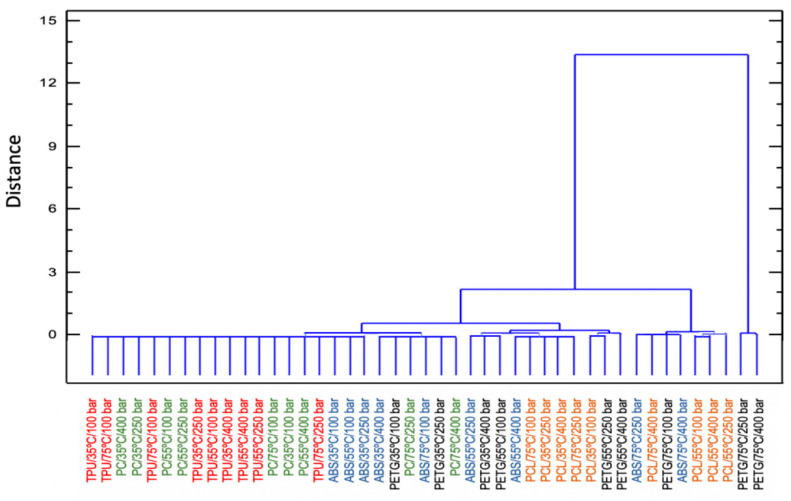
Cluster analysis for the swelling effect of the five polymers under study.

Figure 3 shows the results from the cluster analysis of the swelling achieved from the five polymers under study. The results from the 45 experiments in Figure 1 have been ordered and grouped. There is not a unique criterion to establish the number of items per cluster, but most researchers agree on establishing a relevant difference when there is an abrupt distance between elements, i.e., when the bars become larger in the dendrogram [19]. PETG polymer treated at 75 °C and 250–400 bar of pressure forms a group that corresponds to the maximum swelling of the polymers, reaching values above 200%. ABS at 75 °C and under pressure levels of 250 and 400 bar, PCL at 55 °C at all the studied pressures, as well as at 75 °C and 400 bar and PETG at 75 °C and 100 bar form another group that also corresponds to high swelling percentages in the vicinity of 100%. The rest of the conditions tested are clustered in another subgroup that corresponds to a smaller swelling of the polymers.

Based on this analysis we could conclude that TPU and PETG each represent the group of polymers less affected by the contact with scCO_2_ and the group of polymers most affected by the action of scCO_2_, respectively. Both polymers were selected for the follow-up impregnation studies.

**Figure 4:**
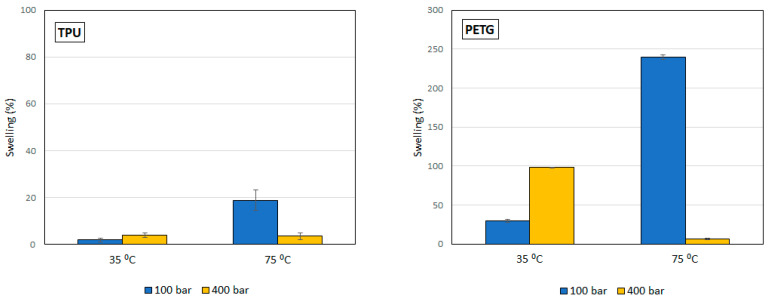
Swelling of the polymers in the supercritical solvent impregnation of MLE.

Figure 4 shows the swelling of PETG and TPU filaments when they have been impregnated with mango leaf extract under different pressure and temperature conditions. A similar behavior to that of the polymer treated only with scCO_2_ can be observed, with a greater swelling, except for the condition of 75 °C and 400 bar, where the swelling when the active ingredient comes into play is less than expected. Table 1 and Table 2 show the results of the statistical analysis of the swelling results obtained for PETG and TPU polymers after the supercritical MLE impregnation process. The ANOVA result indicates that both pressure, temperature and the combined effect of pressure and temperature significantly influence the process (*p*-value lower than 0.05). In PETG swelling, only temperature has a positive effect—an increment of temperature generates an increment in swelling—while pressure and the combined effect have a negative one—an increase generates a decrease in swelling. These variables have a contrary effect in TPU swelling—an increment of pressure generates an increment in swelling, while an increase in temperature, or in the combined effect of both, generates a decrease in swelling.

### 3.2. Impregnation Loading and Antioxidant Activity of the Supercritical Impregnated Polymers

**Figure 5:**
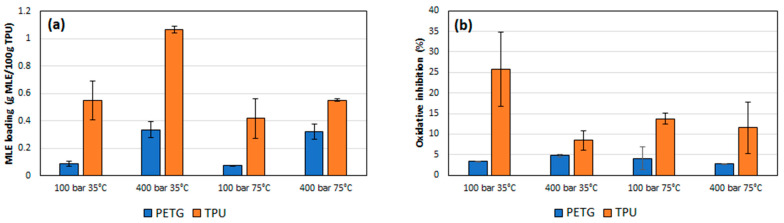
Mango leaves extract impregnation loading (**a**) and antioxidant activity of MLE impregnated polymers (**b**).

Figure 5a shows the MLE loadings by supercritical impregnation under different pressure and temperature conditions of the two selected polymers. Larger loadings were obtained with TPU than with PETG. Regarding the effect of pressure, data shows a positive effect of pressure in both polymers, with best results at 400 bar. The ANOVA tables and the Pareto charts (Table 3 and Table 4) confirm this positive and significant (*p*-value < 0.05) effect of pressure.

Nevertheless, each polymer exhibited a differentiated behavior with respect to temperature. PETG had no relevant differences between the loadings registered at 35 °C and at 75 °C. However, in the case of TPU, while increasing temperature the MLE loading goes down. The ANOVA table and the Pareto charts (Table 3 and Table 4) confirm these observations.

Figure 5b shows the percentage of oxidation inhibition of the impregnated polymers depending on the experimental conditions. As expected, the oxidation inhibition of the impregnated TPU filaments is higher than the PETG ones, since a greater amount of extract had been impregnated into the first polymers compared to the second ones. Regarding the effect of pressure and temperature, again each polymer type presents a different behavior. In the case of TPU, an increase in pressure results in a decrease in antioxidant activity. On the other hand, the effect of the temperature depends on the pressure level, so that it exhibits a lower activity when impregnated at 100 bar and a higher one when produced at 400 bar. This significant (*p* < 0.05) and negative effect of pressure on the polymer’s antioxidant activity was confirmed by the ANOVA table and the Pareto chart (Table 5), while no significant effect could be associated to the different temperature levels. In the case of PETG, the variations of its antioxidant activity associated to temperature or pressure were less significant. Thus, at 35 °C, an increment of the pressure level resulted in an increment of its antioxidant activity, while the same pressure increment when operating at 75 °C led to a loss of its oxidative inhibition properties. On the other hand, when impregnated at 100 bar, an increase in temperature led to an increase in the antioxidant activity of the polymer, while for a 400 bar pressure, the same temperature increment would lead to a reduction of its antioxidant activity. It can be observed from the data in Table 6 that none of the variables have a significant effect on the bioactivity of this type of polymer.

### 3.3. Scanning Electron Microscopy

**Figure 6:**
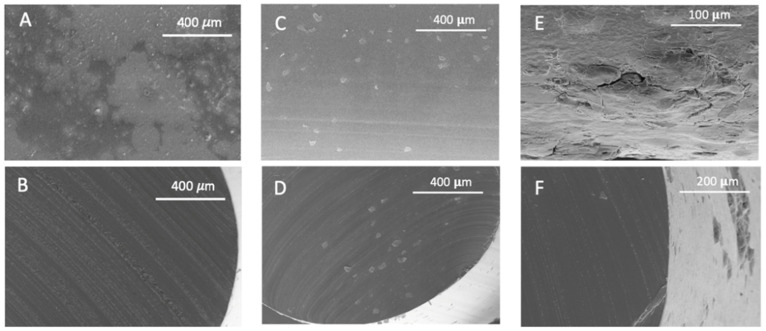
Appearance of raw TPU, (**A**) longitudinal and (**B**) cross section; TPU after scCO_2_ contact (**C**) longitudinal and (**D**) cross section; and TPU after impregnation with MLE, (**E**) longitudinal and (**F**) cross section.

**Figure 7:**
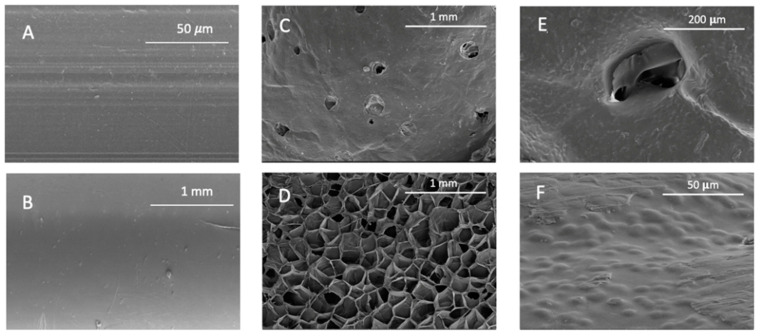
Appearance of raw PETG, (**A**) longitudinal and (**B**) cross section; PETG after scCO_2_ contact, (**C**) longitudinal and (**D**) cross section; and PETG after impregnation with MLE, (**E**) longitudinal and (**F**) cross section.

Figure 6 and Figure 7 show TPU and PETG SEM images of their surface and cross-sections before and after their scCO_2_ treatment and after their MLE impregnation. The scale used is indicated on each picture.

### 3.4. Releasing of the MLE from the Impregnated Polymers

**Figure 8:**
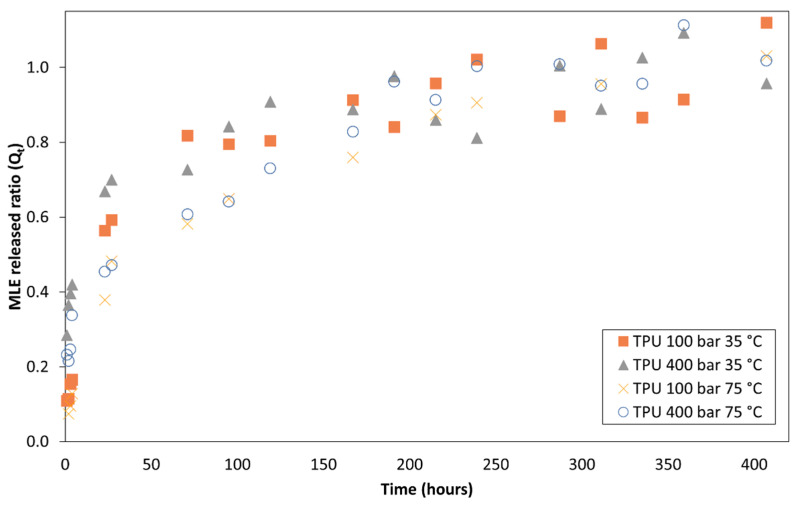
Mango leaf extract release profile from supercritical impregnated TPU at different impregnation conditions.

**Figure 9:**
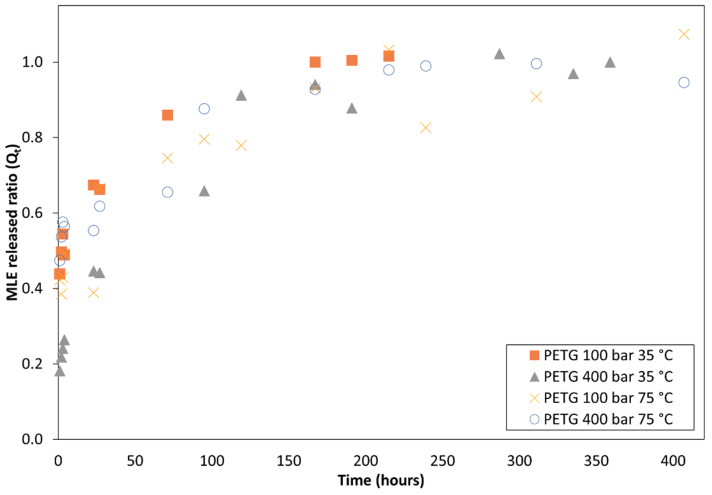
Mango leaf extract release profile from supercritical impregnated PETG at different impregnation conditions.

Figure 8 and Figure 9 show the release profile of the mango extract from the impregnated polymeric samples into a saline medium for about 400 h. Firstly, it should be noted that the release profile was similar for both polymers. A two-stage controlled-diffusion release was observed. During the first moments when the polymer got in contact with the releasing medium, the extract impregnated on the outer surface of the polymer, i.e., around 60% of the total impregnated extract, was quickly released. Subsequently, in a second stage, the extract that had got impregnated inside the polymer gradually diffused through the polymer walls towards the releasing medium. In all the cases, all of the impregnated extract had been released after approximately 200 h of the polymer being submerged into the saline solution. It was also observed that for both polymer types, the samples that had been produced at 75 °C exhibited slightly slower release kinetics than those impregnated at 35 °C.

In order to mathematically model these release profiles, some of the empirical or semi-empirical models that are traditionally used to define the release of drugs from porous matrices were considered. First, the dissolution process of the active compound was considered as a kinetic process and was adjusted to zero-order and first-order kinetics. Second, the experimental data was adjusted to a time-root dependent release through Higuchi equation. Finally, the data were modeled according to the power law by Korsmeyer-Peppas. These equations and the adjusted parameters from each experiment are shown in Table 7. In general, a better fit of the TPU with the Korsmeyer-Peppas equation can be observed according to the data of the R-squared statistic, while the PETG fits better with Higuchi equation.

In all equations $Q_{t}$ is the MLE released ratio at time t (see Equation (7)) and t is time in hours.

## 4. Discussion

The versatility of impregnation processes when using supercritical fluids does not only lie on the advantages of scCO_2_ as a medium to carry substances, but also on the effect that it has on the polymers. Polymers under supercritical conditions modify their structural characteristics and, depending on the operating conditions, this may produce devices that are suitable for different specific applications, which mainly depends on whether or not a process known as “foaming” has taken place during the depressurization phase. When a polymer becomes in contact with carbon dioxide under supercritical conditions, the fluid gets in between the polymer chains and make it turn into a paste as its glass transition temperature decreases. This system reaches a state of oversaturation that causes phase separation and the formation of pores within the polymer matrix. This technique is mainly applied to amorphous polymers, with the exception of polymers with a high crystallinity or glass transition temperature [20].

From the biomedical point of view, the foaming effect may present some advantages depending on the final intended purpose. If the polymer is to be used for tissue regeneration, it should be highly porous, as in the case of scaffolds. A pronounced foaming is quite desirable in this case, as it allows numerous homogeneous and interconnected pores to be generated. These conditions favor cell growth and reduces the rejection of biomedical implants. However, in some particular cases, such as intraocular lenses [21] or in the case of stents [22], an excessive porosity of the polymer would have a negative impact on its functionality. Thus, an increment of the polymer’s porosity would be associated to a higher turbidity of the material, or an excessive cell proliferation that could lead to restenosis. Regarding the use of polymers for food preservation purposes. Again, foaming may have advantages and disadvantages. Thus, when the objective is to produce an active food packaging material, high porosity may affect the mechanical properties of the polymer or let oxygen enter the package. On the other hand, when we intend to use a highly porous material on the inner side of the package, a rather pronounced foaming would be desirable. Therefore, supercritical impregnation processes are to be deeply analyzed and controlled, so that the operating conditions lead to the desired foaming degree according to each specific requirement.

As previously mentioned, according to the results presented in Figure 1, two different behaviors have been observed. Some polymers present a high degree of swelling after being treated with a supercritical fluid. PETG is the most extreme case observed, since it reached a swelling of 250% and suffered structural modifications that can be clearly seen in Figure 7C,D. The nucleation after the impregnation is very evident and even some surface holes became visible. In addition, the porosity of the structures generated with this type of polymers could be modulated and adjusted to the needs of the application for which they are intended by changing the operating conditions of the supercritical impregnation process. At the other end, some polymers barely changed in diameter. For example, TPU had a swelling of 3% in the most drastic conditions of the experiments carried out (400 bar and 75 °C). In fact, when the mild conditions (100 bar and 35 °C) were applied, TPU only modified its diameter by 0.3%. Figure 6C show that the TPU filament that had been treated only with CO_2_ showed a practically even surface and the same can be said of its cross section (Figure 6D). These two polymers were selected to study the supercritical impregnation due to their differentiated behaviors; the extreme cases of the design of experiments were analyzed investigating the effect of the impregnation with MLE.

Regarding the effect of the operative conditions, an increase in the swelling effect was observed as the pressure was increased, at practically all the range of temperatures analyzed. Under isobaric conditions, an increment of the temperature from 35 to 75 °C intensified the swelling effect on all the polymers. As temperature increases, CO_2_ density is reduced and its diffusivity increases. On the other hand, as pressure increases, the solubility of CO_2_ in polymers increases too, even as the diffusion coefficient recedes. Both actions in turn favor the sorption of CO_2_ into the polymer samples and the swelling effect. The absorbed carbon dioxide exerts a plasticizing effect on the polymer that is even more pronounced when the temperature rises over the polymer vitreous transition temperature. At the same time, as the concentration of the fluid inside the polymer increases, its vitreous transition temperature, together with its melting temperature and melt viscosity, significantly decrease. The large swelling and structural deformation observed in the PETG and ABS polymer samples at 400 bar and 55 or 75 °C are a consequence of both effects: plasticization, and the resulting reduction in its melting temperature. In ABS the large deformation is observed above 55 °C, at all the pressures studied. Other studies can be found in the literature, where the same behavior was reported. Thus, Verano-Naranjo [17] studied the swelling effect on PLA in contact with scCO_2_ and registered volume increments as pressure was increased (from 100 to 400 bar) and as temperature was increased from 35 to 75 °C. On the other hand, Champeau and coworkers [23] also observed an increase both in the sorption of CO_2_ and the degree of swelling of the PLLA fibers as pressure was increased up to 150 bar at 40 °C temperature.

Concerning the swelling observed under the different conditions of the MLE impregnation experiment, both impregnated polymers have the same behavior at 35 °C. An increase in pressure favors swelling as a consequence of the sorption of scCO_2_ into the polymer sample. However, a smaller swelling than expected was obtained for the TPU and PETG samples impregnated at 400 bar and 75 °C. Under supercritical conditions, the interaction between the CO_2_ and the ethanol disrupts any predictable solvent’s density, as reported by Pöhler and Kiran [24]. This phenomenon could explain why unpredicted swelling may occur. In addition, the MLE can modify the vitreous transition temperature of the polymers.

When the MLE was impregnated into both of the polymers selected, the loading of MLE varied greatly depending on the type of polymer. The much larger loadings were achieved by TPU at nearly 1.1 mg of MLE per 100 mg of polymer when processed at 400 bar and 35 °C. Zhang et al. [25] studied the effect of supercritical carbon dioxide on the loading of different drugs on TPU films impregnated at 150 bar and 40 °C. They reported a maximum loading of 1.56 µg/mg of 7-hydroxycoumarin. The amounts of MLE impregnated in the present work for similar conditions (100 bar and 35 °C) are higher than those reported by Zhang’s study, which seems to indicate a closer affinity of MLE with the polymer and with CO_2_ than the drug employed by Zhang.

It can be seen from the SEM images of the TPU filaments that MLE had deposited on the surface of the polymer (Figure 6F), which indicates the possibility of a superficial impregnation. In fact, no differences in the internal part of the filament can be inferred from the Figure 6D,F. However, PETG seems to be a quite different case, since its high porosity would allow the penetration of the extract into the polymer and hardly any superficial coating can be observed (Figure 7E,F). The polymer presents about 10 micron bulges formed by the extract on the surface and on the walls of the holes generated by the foaming phenomenon. A similar aspect of mango extract has already been observed in other polymers, such as cotton [26]. Nevertheless, PETG does not achieve such large loadings, which could be partly explained by the fact that that CO_2_ probably drags away larger amounts of the extract compounds during the depressurization phase, given that the pores generated on the surface and inside the polymer have a longer diameter of around 200 μm (Figure 7).

There are many factors in the supercritical impregnation process: the physicochemical interactions between the three components of the process—the solute (MLE), the polymer and the scCO_2_ phase—as well as the effect of operating parameters such as pressure, temperature, contact time, depressurization rate and solute-matrix ratio. Specifically, pressure and temperature play an important role in the impregnation process since these two parameters have a significant influence on the solubility of the active substance into the CO_2_ phase. In general, an increase in pressure increases the CO_2_ density, together with its swelling effect [27], and favors the solubility of the MLE in the supercritical phase, which results in a greater amount of MLE saturated CO_2_ going into the polymer structure and, therefore, a greater MLE loading. This positive effect of pressure was more noticeable for TPU at 35 °C. Regarding temperature, while TPU achieves higher loads at 35 °C, there is hardly any influence of the operating temperature on the extract loaded in PETG. An isobaric increment of temperature causes a decrease in CO_2_ density and in its transport properties, which negatively affects its impregnation efficiency. On the other hand, a higher temperature increases the vapor pressure of the active compounds. Therefore, since both effects are opposite, only by experimental study can we find out which one is the predominant factor in each polymer.

Despite the lower loadings achieved by PETG when compared against those exhibited by the TPU samples, their antioxidant capacity is not that different. This is explained by a much more efficient release of the MLE into the medium, while many of the MLE compounds of interest remained attached to the surface of the TPU. When comparing against the results obtained by Rosales et al. [8] who applied the same conditions to impregnate polylactic acid (PLA) with mango leaves extract, we can observe that the inhibition percentage reported after 9-day incubation periods was just 9.9 ± 1.1% against the 25.75 ± 0.09% of the MLE-impregnated TPU achieved by this assay. The way that the extract substances, either antioxidant or non-antioxidants, compete during the impregnation process, changing as the operating conditions are also modified. Such competition is produced between the solubility of the compound in the supercritical phase and the retention in the polymer. There is no direct correlation between the loading and the antioxidant activity of the impregnated polymer. Hence, increasing pressure from 100 to 400 bar at 35 °C for TPU impregnation decreases the antioxidant activity of the sample due to non-antioxidant substances were impregnated.

About the release kinetics of the impregnated extract, the two polymers exhibit a two-phase diffusion process, as already explained in the results section (Figure 8 and Figure 9). Under all the impregnation conditions tested the release curve was quite similar. A slightly lower velocity could be observed by the samples of both polymers when impregnated at 75 °C compared to those impregnated at 35 °C. Nevertheless, the impregnation conditions do not seem to affect the release mechanism. The rate at which the active compound particles reach the diffusion medium depends on a number of factors. One of them is the depth at which the compounds have been absorbed into the porous matrix. It is to be expected that under a higher impregnation pressure and temperature the MLE molecules would penetrate deeper into the matrix and therefore their subsequent release kinetics would be slower. This would also be influenced by the swelling degree of the polymer filament during its impregnation, since a greater swelling would allow a deeper penetration of the MLE compounds. This could, therefore, explain why the samples of both polymers that had been impregnated at 75 °C, and achieved a greater swelling, exhibit slightly slower kinetics than those impregnated at 35 °C.

The release kinetics of polymeric matrices also depends on the biodegradability of the polymer matter. In the case of biodegradable polymers, their degradation is expected to start at some point during the release process, which would result in an increment of the release rate of the impregnated compounds. In our case, although TPU is a biodegradable polymer, its release curve does not present any changes. This probably indicates that the degradation process is slower than the release of the compounds. On the other hand, since PETG is a non-biodegradable polymer its release curve presents a progressive slope that decreases over time, without any significant changes in the release rate.

Regarding the mathematical model (Table 7), it could be verified that the data do not fit so well to zero and first-order models, with better fit to zero order of PETG kinetics, while TPU’s fit better to first-order models. In fact, mathematical models are best applied to biodegradable polymeric systems where the degradation kinetics of the material matches the dissolution kinetics of the active compound, but this is not our case for either type of polymer tested. Nevertheless, a better fit was obtained for both polymers to Higuchi model based on Fick’s law, which suggests a diffusional release. Other authors have reported a time-dependent root release for these polymers; for example, Welsh and coworkers [28], found this type of release profile in TPU vaginal rings containing dapivirine, a microbicide intended to prevent certain sexually transmitted diseases. On the other hand, the fit to the power law was quite good and an exponent of the equation values lower than 0.45 were observed. This implies a normal or Fickian diffusional release according to the values of this exponent as reported by Ritger and Peppas [29] for polymeric devices with cylindrical geometry.

## 5. Conclusions

The pharmaceutical industry is currently investigating new methods to control the release, and therefore the dosing, of active substances intended for the treatment of different medical disorders. The functionalization of polymeric objects through their supercritical impregnation is one of the effective approaches towards this goal, since they have proven to exhibit the desired bioactivity conferred by the active substance they are impregnated with. Nevertheless, the different behavior exhibited by the different types of polymers when subjected to supercritical impregnation determine their suitability for each specific medical application.

Some promising results have been obtained towards the production of materials that can have a practical use in biomedical or food industry. Thus, by varying pressure and temperature conditions, the effect of supercritical carbon dioxide on some polymers can be modulated. This is rather evident when processing PETG, ABS or PCL samples, while for other polymer types, such as PC or TPU, this effect is not so obvious.

When the material to be produced requires a high porosity, like in the case of scaffolds, aerogels or foams to be incorporated to food containers, PETG impregnated under moderate or high pressure and temperature conditions seems to be the best choice. If, conversely, the objective is to produce a material with a low porosity to be used for stents or food preserving packing films, either TPU or PETG impregnated at low pressure and temperature conditions are the most suitable options.

## Figures and Tables

**Table 1:** ANOVA table and Pareto chart for quadratic model of swelling of PETG in the SSI with MLE.

	Sum Sq	Df	Mean Sq	F-Value	*p*-Value	
**A: Pressure**	27,077.5	1	27,077.5	17,300.51	0.0000	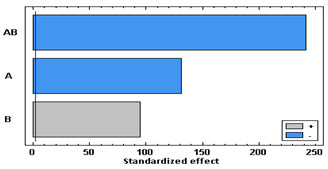
**B: Temperature**	13,976.6	1	13,976.6	8929.98	0.0000	
**AB**	91,205.5	1	91,205.5	58,273.50	0.0000	
**Blocks**	30.7636	3	10.2545	6.55	0.0122	
**Error**	14.0862	9	1.56513			
**Total**	132,304	15				
R-square = 99.9894%						

**Table 2:** ANOVA table and Pareto chart for quadratic model of swelling of TPU in the SSI with MLE.

	Sum Sq	Df	Mean Sq	F-Value	*p*-Value	
**A: Pressure**	179.773	1	179.773	52.10	0.0000	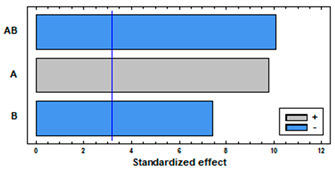
**B: Temperature**	276.703	1	276.703	80.19	0.0000	
**AB**	298.553	1	298.553	86.52	0.0000	
**Blocks**	36.8652	3	12.2884	3.56	0.0604	
**Error**	31.0545	9	3.4505			
**Total**	822.95	15				
R-square = 96.2264%						

**Table 3:** ANOVA table and Pareto chart for quadratic model of MLE loading in TPU.

	Sum Sq	Df	Mean Sq	F-Value	*p*-Value	
**A: Pressure**	0.210601	1	0.210601	24.64	0.0157	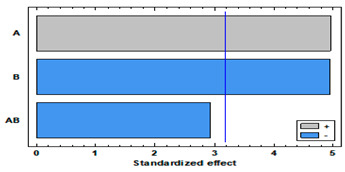
**B: Temperature**	0.20911	1	0.20911	24.47	0.0159	
**AB**	0.0734594	1	0.0734594	8.59	0.0609	
**Blocks**	0.0162	1	0.0162	1.90	0.2623	
**Error**	0.0256409	3	0.00854696			
**Total**	0.535011	7				
R-square = 95.2074%						

**Table 4:** ANOVA table and Pareto chart for quadratic model of MLE loading in PETG.

	Sum Sq	Df	Mean Sq	F-Value	*p*-Value	
**A: Pressure**	0.1235	1	0.1235	82.46	0.0028	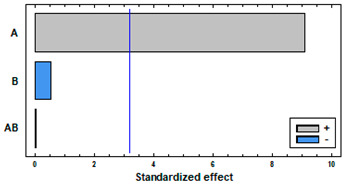
**B: Temperature**	0.0004	1	0.0004	0.28	0.6329	
**AB**	0.0002	1	0.0002	0.00	0.9731	
**Blocks**	0.0023	1	0.0023	1.54	0.3023	
**Error**	0.0045	3	0.0015			
**Total**	0.1307	7				
R-square = 96.5632%						

**Table 5:** ANOVA table and Pareto chart for quadratic model of antioxidant activity of impregnated TPU.

	Sum Sq	Df	Mean Sq	F-Value	*p*-Value	
**A: Pressure**	377.525	1	377.525	13.19	0.0055	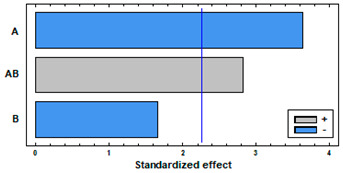
**B: Temperature**	79.0321	1	79.0321	2.76	0.1310	
**AB**	228.161	1	228.161	7.97	0.0199	
**Blocks**	128.538	3	42.8461	1.50	0.2805	
**Error**	257.622	9	28.6247			
**Total**	1070.88	15				
R-square = 75.9429%						

**Table 6:** ANOVA table and Pareto chart for quadratic model of antioxidant activity of impregnated PETG.

	Sum Sq	Df	Mean Sq	F-Value	*p*-Value	
**A: Pressure**	1.4981	1	1.4981	0.56	0.4737	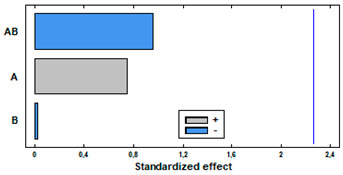
**B: Temperature**	0.0012	1	0.0012	0.00	0.9834	
**AB**	2.4539	1	2.4539	0.92	0.3636	
**Blocks**	16.434	3	5.4780	2.04	0.1782	
**Error**	24.113	9	2.6793			
**Total**	44.501	15				
R-square = 45.8134%						

**Table 7:** Mathematical models for MLE release.

Model	Sample	Model Parameters	Adjusted Parameter
Zero-order $Q_{t}=k_{o}t$	TPU 100 bar 35 °C	$k_{o}=0.002$	$R^{2}=0.675$
	TPU 400 bar 35 °C	$k_{o}=0.003$	$R^{2}=0.833$
	TPU 100 bar 75 °C	$k_{o}=0.005$	$R^{2}=0.782$
	TPU 400 bar 75 °C	$k_{o}=0.002$	$R^{2}=0.860$
	PETG 100 bar 35 °C	$k_{o}=0.003$	$R^{2}=0.894$
	PETG 400 bar 35 °C	$k_{o}=0.002$	$R^{2}=0.804$
	PETG 100 bar 75 °C	$k_{o}=0.002$	$R^{2}=0.759$
	PETG 400 bar 75 °C	$k_{o}=0.001$	$R^{2}=0.775$
First-order $Q_{t}=\exp\left(k_{1}t\right)$	TPU 100 bar 35 °C	$k_{1}=0.004$	$R^{2}=0.536$
	TPU 400 bar 35 °C	$k_{1}=0.005$	$R^{2}=0.741$
	TPU 100 bar 75 °C	$k_{1}=0.013$	$R^{2}=0.671$
	TPU 400 bar 75 °C	$k_{1}=0.004$	$R^{2}=0.750$
	PETG 100 bar 35 °C	$k_{1}=0.004$	$R^{2}=0.833$
	PETG 400 bar 35 °C	$k_{1}=0.114$	$R^{2}=0.833$
	PETG 100 bar 75 °C	$k_{1}=0.065$	$R^{2}=0.791$
	PETG 400 bar 75 °C	$k_{1}=0.049$	$R^{2}=0.837$
Higuchi $Q_{t}=k_{H}\;t^{1/2}$	TPU 100 bar 35 °C	$k_{H}=0.047$	$R^{2}=0.847$
	TPU 400 bar 35 °C	$k_{H}=0.049$	$R^{2}=0.944$
	TPU 100 bar 75 °C	$k_{H}=0.071$	$R^{2}=0.887$
	TPU 400 bar 75 °C	$k_{H}=0.045$	$R^{2}=0.963$
	PETG 100 bar 35 °C	$k_{H}=0.042$	$R^{2}=0.976$
	PETG 400 bar 35 °C	$k_{H}=0.048$	$R^{2}=0.939$
	PETG 100 bar 75 °C	$k_{H}=0.035$	$R^{2}=0.876$
	PETG 400 bar 75 °C	$k_{H}=0.029$	$R^{2}=0.893$
Power law $Q_{t}=k_{P}\;t^{n}$	TPU 100 bar 35 °C	$k_{p}=0.112;\;n=0.397$	$R^{2}=0.941$
	TPU 400 bar 35 °C	$k_{p}=0.306$; n=0.218	$R^{2}=0.984$
	TPU 100 bar 75 °C	$k_{p}=0.056$; n=0.565	$R^{2}=0.964$
	TPU 400 bar 75 °C	$k_{p}=0.200$; n=0.278	$R^{2}=0.977$
	PETG 100 bar 35 °C	$k_{p}=0.428$; n=0.159	$R^{2}=0.973$
	PETG 400 bar 35 °C	$k_{p}=0.175$; n=0.308	$R^{2}=0.986$
	PETG 100 bar 75 °C	$k_{p}=0.361$; n=0.167	$R^{2}=0.841$
	PETG 400 bar 75 °C	$k_{p}=0.463$; n=0.124	$R^{2}=0.864$

## Data Availability

All the data presented in this study are available in this article.
